# Enhancing Equality, Equity, Diversity and Inclusion in Rare Disease Research in the United Kingdom

**DOI:** 10.3390/nursrep15100361

**Published:** 2025-10-09

**Authors:** Andrew E. P. Mitchell, Sondra Butterworth

**Affiliations:** Faculty of Health, Medicine and Society, University of Chester, Wheeler Building, Castle Drive, Chester CH1 1SL, UK; s.butterworth@chester.ac.uk

**Keywords:** equality, equity, diversity, inclusion, rare disease, nursing, public and patient involvement, recruitment, mixed methods, representative findings

## Abstract

**Background**: Inclusion of under-represented rare-disease communities in research remains limited, threatening representativeness and equity. **Methods**: To assess equality, equity, diversity, and inclusion in research and identify barriers to participation faced by the rare disease community, utilising a mixed-methods online survey of a convenience sample of community advocates using Likert scales and free response options. **Results**: The findings from seventeen stakeholders in the rare disease community showed unanimous agreement that anxiety, fear, safety concerns, and lack of trust hinder participation in research. A total of 82% agreed or strongly agreed that additional financial resources are needed, and 76% agreed or strongly agreed that research grant applications often lack sufficient funds. The free-text responses demonstrate that the rare disease communities are keen to be involved in research but faces barriers to inclusion. Rare disease communities are willing to participate in research, but those responsible for research need to address the challenges related to language, misconceptions and fear. **Conclusions**: Key legislation in the United Kingdom, specifically the Proposed Patient and Public Involvement Strategy 2020–2025, emphasises the importance of involving patients and the public in health and social care. This survey marks the first step toward gaining valuable insights into the challenges faced by this community in participating in healthcare research, which is crucial for developing a solid evidence base for their treatment and care. Involving stakeholders is essential in health and social care policy and practice, rooted in advocacy and social justice.

## 1. Introduction

The issues of equality, equity, diversity, and inclusion in rare disease research have been highlighted, especially for the involvement of the rare disease community [1]. This paper addresses rare disease communities and the diversity amongst this group and their inclusion in research. Equality, lack of diversity, and inclusion in rare disease research, especially clinical studies, have been extensively documented [2]. Multiple factors, including the intersection of health inequalities and rare disease symptoms, result in low levels of diverse participation, hindering comprehension of new treatments’ safety and efficacy [3]. Studies on rare diseases extend beyond clinical trials to include investigations of health disparities, a United Kingdom (UK) and international scoping review of 136 studies [4], surveying patient experiences to highlight a gap in rare disease frameworks of ‘trust and confidence in healthcare professionals’ [5], and key policy analysis and key initiatives to create a supportive legislative environment for rare disease patients focusing on enhancing research and development to better meet the needs of those affected by rare diseases [6].

Often, we hear the acronym EDI, or in rare disease research, the acronym DEI, which is used to represent three terms: diversity, equity, and inclusion. EDI and DEI share the same elements, diversity, equity, and inclusion. However, DEI emphasises representation and prioritises diversity. These two acronyms do not include the term equality. Equality addresses structural barriers that hinder individuals from reaching their potential, while diversity and inclusion work together to create environments where individuals from various backgrounds are not only represented but also actively integrated and valued for their unique perspectives, fostering social justice and meaningful change [3]. It is becoming more common to mention equity with equality, as treating everyone the same may not guarantee equal opportunities or outcomes. While equality, diversity, and inclusion (EDI) are essential, it is crucial to prioritise equity. Equity acknowledges that individuals do not start from the same point and may need extra resources or opportunities to achieve equality, rather than just treating everyone the same. Therefore, equality, equity, diversity, and inclusion (EEDI) will be used as the preferred term.

The Rare Diseases Framework of 2021 [7] is a piece of United Kingdom (UK) policy, focusing on a “national vision on how the UK will improve the lives of those living with rare diseases” [7] (p. 2). The Framework defines a rare disease as “… a condition which affects less than 1 in 2000 people. It is currently estimated that there are over 7000 rare diseases, with new conditions continually being identified as research advances” [7] (p. 2). The Framework expands on the definition by indicating that “rare diseases are individually rare, they are collectively common, with 1 in 17 people being affected by a rare disease at some point in their lifetime. In the United Kingdom, this amounts to over 3.5 million people. It is therefore important that the NHS and other services provide this large and diverse patient population with the best possible care” [7] (p. 2).

This study contains the survey results administered to seventeen members representing smaller charities and community advocates from the rare community disease network and the Equality, Diversity, and Inclusive Research Association (EDIRA). Charities and community advocates play a crucial role in the rare disease community. These partner organisations served as gatekeepers for the present study. They provide educational resources and support for patients and their families, while also working to advance policies, research, and therapeutic development for rare conditions. It is important to acknowledge that patients with rare diseases face many challenges due to their condition. They often have to rely on self-advocacy and advocacy from others to advance clinical research for their diseases. In recognition, local contacts with charities and community advocates were contacted for support and co-design of the survey questions. It is recognised that empowerment of underserved communities and engagement can help address broader inequities [8].

The survey was an online EEDI questionnaire aiming to explore opinions and views related to EEDI in research and research ethics within the rare disease community, which could be used to identify barriers and obstacles. Due to the infrequency of their disease, individuals with rare medical conditions often face significant difficulties in terms of diagnosis, research, and clinical care. These challenges are often observed regardless of the type or prevalence of the condition. The objective is to recognise barriers to diversity, equity, and inclusion and pinpoint the barriers that impede participation and research ethics. This information can help shape EEI practices and initiatives in rare diseases.

The survey details the perspectives and experiences of those working in the field regarding diversity, equity, and inclusion in research access in the rare disease community. The survey reveals their perspectives on research and the research process within the rare disease community and their responses to equality, equity, diversity, and inclusion challenges.

## 2. Materials and Methods

An online survey was developed iteratively, incorporating feedback from those working in the rare disease community. The convergent mix method design [9] includes both closed-ended, Likert scale, and open-ended questions. The survey was uploaded onto the JISC Online survey tool [10] and IP addresses were not collected. Genomics Partnership Wales patient sounding board, the Rare Quality of Life and its Rare Community Network, and EDIRA were aware of the survey. They consented to receiving and distributing the online link for the survey. Ethical approval was provided by the Faculty of Health, Medicine and Society Research Ethics Committee (RESC0722-1109). Data were stored electronically, and all data were anonymised and collected under the principles of the Declaration of Helsinki [11], the legal requirements for the UK Data Protection Act [12], and the University’s Research Governance requirements. Data collection occurred from mid-November 2002 to mid-January 2023, with informed consent obtained through a consent form and a participant information sheet.

The Section 1 contained demographics, i.e., sexual orientation and gender identity (SOGI) questions, age, and rare disease conditions about self or family members. The format adhered to good practice guidelines for SOGI [13] questions were followed and any questions that did not help enhance understanding were not included in Table 1 to protect anonymity and to succinctly report demographics that enriched the analysis, i.e., sexual identity and religion.

The Section 2 of the questionnaire had nine Likert questions on a five-point scale, as reported in Table 2. A stakeholder from the rare disease community developed the short survey with knowledge of the area. While the questions may focus on potential issues, the lead author highlighted the need to maintain the developed questions for authenticity, to support content validity of the stakeholders. The questions also underwent a pilot to improve their reliability and effectiveness. The lead author’s role was to analyse and report the survey data. The survey also included definitions for complex terms, such as “intersectionality,” which is defined as “the impact of an individual’s characteristics and the potential for inequality.”

The convenience sample was utilised as the three stakeholder organisations, Genomics Partnership Wales patient sounding board, the Rare Quality of Life, and its Rare Community Network, were members of EDIRA and were contactable through the association. It was recognised that this might lead to a potential selection bias. A sampling frame from these stakeholder organisations represented an estimated pool size of 56 contacts. These partnerships also facilitated requests for anonymous assistance to help address inquiries or provide specific support regarding survey completion, rather than having participants contact the first author directly. Additionally, they made a final call and reminder for participants to complete the survey. Both strategies were implemented to enhance the response rates of the survey. No incentives were offered.

The members of the association thought a short survey was most appropriate due to cost and time, and while COVID-19 is no longer a public health emergency, it still remains a technical pandemic, and vulnerable communities could still be at risk with face-to-face interviews. A short survey was also considered a discreet way to gather insights into the unique experiences, challenges, and needs of individuals living with rare diseases. The format allowed collection of insights and opinions, making participants feel more comfortable sharing their thoughts and participating in the process. Furthermore, collecting data through surveys supports evidence-based decision making by facilitating consensus measurements.

Therefore, a short online survey was developed with a number of stakeholder categories in mind with a view to building upon any findings with the community as co-participants. During two consultation meetings organised by EDIRA, key issues and components were identified. The choice not to conduct formal validity or reliability testing was made because the focus was on exploring concepts and gathering diverse perspectives rather than ensuring precise measurements. The goal was to identify themes or patterns for further investigation. A small pilot with no conditional branching was conducted to address any issues with the survey before its upload to the JISC survey tools.

## 3. Results

The use of the SOGI questions in the survey was important in identifying this community’s demographics and potential disparities. The completion rate for all fixed questionnaires was 100%, due to the electronic design, which included an option for “prefer not to say” for all fix response questions. The reporting was restricted to protect individual anonymity. Some endorsements could not be included due to either N = 1 and a correlation between categories of sexual orientation and gender identity, which would risk anonymity in a small sample. Therefore, this information is not presented in Table 1. Researchers and practitioners should be intentional in their approach to asking and reporting SOGI questions, as this can help understand and addresses health disparities [14].

### 3.1. Characteristics of Participants

There were five categories: patient group (29.4%), advocates (23.5%), charity or third sector (17.7%), National Health Service (5.9%), and others (23.5%), including academics working in higher education, member organisations of a patient group, and a consultant and researcher, all of which offer services to the community of people living with rare diseases.

Seventeen members of the rare disease community participated in the survey. There were 11 female participants (64.7%), five male participants (29.4%), and one non-binary participant (5.9%). Most participants (41.2%) were aged between 40 and 50. Table 1 reports the main participant demographics and gender identity endorsements.

Five of the seventeen participants (29.4%) reported living with a rare condition themselves and five (29.4%) reported having a family member with a rare condition. The five participants reporting with having a rare condition but were also working either as paid or unpaid roles within the rare disease community and decision-making capacity was not seen as an issue for these participants. Two participants disclosed their rare condition, and two family members conditions were named. The participants’ rare condition and frequency are listed in Table 1.

Those reporting rare conditions have been categorised to protect privacy as having genetic blood disorder, muscular disorder, tumour, connective tissue, or skin disorder. One participant declined to identify their rare condition, while another failed to disclose.

Participants identified family members with rare disease diagnoses, including four genetic syndromes, two neurodegenerative diseases, and one cardiovascular condition. Two participants chose not to identify their rare condition, while two others failed to mention the condition.

#### 3.1.1. Consideration of the Economic Situation

The Likert scale interpretation intervals and confidence intervals (CIs) are utilised to summarise the endorsements. The CIs reflect the degree of consensus within the data. When the CIs overlap with the Likert interpretation intervals, they are presented and interpreted accordingly with the strength of the scaled agreement or disagreement, i.e., strongly agree to strongly disagree.

Table 2 shows opinions of economic, diversity, equity, and inclusiveness barriers to perceived participation in research studies. The term participation was for involvement at any stage in the research process, including conception, assessment, analysis, dissemination, and evaluation. The nine questions on the barriers to participation in research can be found in Table 2.

Fourteen participants (82.3%) indicated on question one that they either strongly agreed or agreed that the lack of monetary resources was a barrier to participation in research. The participant category mode with the highest frequency of responses, ‘advocates’ was for ‘strongly agree’. The second question in this section looked at researcher grant applications and monies available to pay for participants’ out-of-pocket expenses or costs to participate in the research.

Thirteen participants (76.4%) either strongly agreed or agreed that funding applications often lacked sufficient provision for these expenses, including those from the rare disease communities. The participant category mode with the highest frequency of responses, ‘charities, patient groups and others in education or research,’ was for ‘strongly agree’.

The third question examined the understanding of intersectionality and the impact intersectionality has on inclusion in research. The consideration of intersectionality is about understanding and widening participation for meaningful involvement in research. Thirteen participants (76.4%) either strongly agreed or agreed that a lack of understanding of intersectionality was an issue for meaningful inclusion in research.

#### 3.1.2. Equality, Equity, Diversity and Inclusiveness Considerations

The fourth question was about gathering viewpoints on recruiting participants from diverse groups, as shown in Table 2. Fourteen participants (82.3%) indicated that they either strongly agreed or agreed that researchers were often unsure how to access participants from diverse groups. The participant category mode with the highest frequency of responses, ‘charities and others (education and researchers),’ was for ‘strongly agree’ and 11.8% of participants disagreeing. The next question looked at participation in those who could not potentially consent and the exclusion of those lacking mental capacity. Fifteen participants (88.2%) either strongly agreed or agreed that studies do not consistently demonstrate how to include participants who may lack capacity. The participant category mode with the highest frequency of responses, ‘charity’, was for ‘strongly agree’ and 11.8% of participants disagreeing.

The sixth question in this section looked at cultural understanding. Twelve participants (70.5%) either strongly agreed or agreed that a researcher’s understanding of cultural perspective was a barrier to participation in research. The participant category mode with the highest frequency of responses was ‘advocates and others (education and researchers)’, for ‘strongly agree’.

Language was the focus for the next question, with sixteen participants (94.1%) agreeing or strongly agreeing that complicated language or the absence of translation services in recruitment impedes inclusiveness in research. The participant category mode with the highest frequency of responses, ‘patients or patient groups’, was ‘agree’. The next question was on how disability was perceived. Thirteen participants (76.4%) agreed or strongly agreed that the medical model was the dominant perspective over the social model of disability. The participant category mode with the highest frequency of responses, ‘advocates’, was for ‘strongly agree’. A total of 11.8% of participants disagreed, and another 11.8% were uncertain that this was an issue.

In the final question, seventeen participants (100%) agreed or strongly agreed that anxiety, fear, safety, and lack of trust are barriers to research participation. The participant category mode with the highest frequency of responses, ‘patients or patient groups’, was ‘agree’. This reiterates the dominant viewpoint, further emphasising its significance.

### 3.2. Qualitative Responses and Themes

The analysis of free-text responses to reflections on the inclusiveness of research and research ethics yielded qualitative two themes ensuring reflexivity and analytic depth by and following a framework [15]. The process began with familiarising ourselves with the data, followed by generating initial codes to categorise meaningful segments using reflexive thematic analysis. The first author manually searched for themes, organising the codes into broader patterns and reviewing these themes to ensure accuracy and coherence. Due to the exploratory nature of the study, no participant checking was conducted; however, a joint, integrated data collection approach for convergent mixed methods was used to enhance credibility. The final step involved compiling the findings and creating a composite table (Table 3) with both quantitative and qualitative data.

#### 3.2.1. Accessibility and Language in Research

The most challenging aspects are the practical aspects of inclusion in research that affect diversity in participation.

“I have never worked at, or even heard about, a research institution that provided language translation services to enable research. Puzzlingly, many of these institutions also teach languages.” Quote from participant with a rare condition, 40–50 age group, male.

And another participant similarly mentions:

“… all included in the re-search process to ensure these voices are heard. I believe this type of research seeks to take these voices into the overall conversation and research design.” Quote from an advocate with a rare condition, 40–50 age group, female.

According to individuals with a rare disease and advocates in the community, research goals must encompass clear definitions of diversity, inclusion, and equity to increase participation from underserved groups.

“Research which aims to take into consideration the diversity of the population and aims to include underrepresented groups …” Quote from patient or patient group, no rare condition, 40–50 age group, male.

“Research that involves stakeholders from a diverse pool of humans, i.e., ethnically diverse, racially diverse, diverse gender-identities, diverse economic circumstances, and other forms of human diversity.” Quote from advocate, with a rare condition, 40–50 age group, female.

The key takeaway from these verbatim opinions on this theme is that research should be accessible to all sections of society, consider diversity, include underrepresented groups, involve participants throughout, and engage with diverse stakeholders.

#### 3.2.2. Tensions Involved in Achieving Participation

The responses demonstrate that the rare disease community is keen to be involved in research but faces barriers to inclusion, including respect, trust and misconceptions. The free-response data from participants also mentioned that involvement in research must span from planning to dissemination.

“Research that involves and considers participants in the design, deliver and monitoring and evaluation through-out” Quote from an advocate with a rare condition, 62–72 age group, female.

Another participant mentioned the importance of diversity in research, specifically involving individuals from underrepresented groups.

“Research which aims to take into consideration the diversity of the population to study and aims to include underrepresented groups in the study.” Quote from a patient or patient group, no rare condition, 40–50 age group, male.

The rare disease community members proposed ways to improve rare disease participation in research. Some emphasised the importance of including patient voices, while others in education and research highlighted the need to address the challenges related to language, misconceptions and fear.

“To include a cross section of whole community, to ensure all voices are heard” Quote from a patient or patient group, no rare condition, 51–61 age group, female.

“Research which aims to take into consideration the diversity of the population to study and aims to include underrepresented groups in the study.” Quote from a patient or patient group, no rare condition disclosed, 40–50 age group, male.

“Fears of harm, fear of stigma, fear of unemployment, fear of side effects, these are all very real.” Quote from an advocate, no rare condition, 51–61 age group, female.

The quotes in this theme highlight the need to involve and consider participants in the design, delivery, monitoring, and evaluation of research and remove barriers to participation. Finally, these quotes mention the importance of participatory research involving those not generally reached by traditional research approaches.

## 4. Discussion

The findings from the present research highlight views and opinions from the rare disease community on equality, equity, diversity, and inclusion in research and, importantly, the barriers to accessing research participation. The relevance of nursing practice will be established through existing literature and relevant policies in the UK and internationally. Nurses, with their understanding of the social aspects of healthcare systems and training in scientific research, may be strategically positioned. The findings suggest that researchers could do more to ensure that the rare disease community is represented in research and that equality, equity, diversity, and inclusion are valued. For the rare disease community, a lack of financial resources was perceived as a barrier to research participation. Researchers were perceived to need help to fully appreciate the challenges intersectionality posed for the rare disease community, and this was viewed as a significant barrier.

There was acceptance of obstacles based on economic resources, but the lack of monetary resources was a barrier to participation in research, with the highest frequency of responses arising from ‘advocates’ and overall finding from participants were agree or strongly agree. The second reflection area looked at grant applications and monies available to pay for participants’ out-of-pocket expenses or costs. The mode participant category with the highest frequency of responses was from ‘charities, patient groups and others working in education or research’. It showed ‘agree’ and ‘strongly agree’ (question 2 on Likert scale) that additional financial resources were needed for participation in research. This aligns with the findings of a UK study that found payment attracted more socioeconomically disadvantaged participants [16,17] and was enhanced by incentive payment to enable research participation. The well-known costs include costs associated with participation are transportation, extra time adjustment, and support needed from a carer to enable them to travel in some cases and additional equipment necessary for involvement [18]. These opinions support previous research looking at the undesirable implications of the lack of economic support to meet the costs incurred by participants in clinical trials [19,20]. The cost is likely to increase the cost of research but is expected to be modest in comparison to the cost of a research project as a percentage [21].

There was a finding from the present study regarding the cultural, linguistic barriers, and capacity to consent to participation in research as ‘agree’ and ‘strongly agree’ (question 2 Likert) and qualitative theme, accessibility, and language in research (Table 3; upper component of the composite table). This aligns with a UK study looking at cultural and linguistic barriers [22] by using bilingual interpreters. Another UK study focused on impaired capacity to consent [23] by using a consent support tool. Both of these studies showed that communication between researchers and participants to supports inclusion and diversity. The approach can be seen in international initiatives such as Lyfe Languages, which addresses communication issues related to rare diseases, builds supporting narratives, and creates trust [24].

There was also a general agreement that anxiety, fear, safety concerns, and lack of trust are obstacles to research participation. A UK study highlights issues in underserved communities [25], emphasising that trust is a key factor for engagement. This trust can be enhanced by creating a safe space and rebuilding partnerships but has not always been observed. For example, diverse rare disease communities are often excluded from research and challenged by a legacy of fear and mistrust in response to exploitative “helicopter research” [26], which refers to a type of study where researchers briefly engage with a community without fully understanding its context or forming meaningful relationships. It involves collecting data and then leaving [27]. Building trust and creating a safe space will likely be achieved only by engagement, co-production, and trust [28]. This positions nurses, due to their expertise in healthcare and social care systems, to uniquely advocate for equitable access and address disparities in underserved communities [29].

While a few findings in the present study could have been more persuasive regarding their mention and endorsements, they are still important to report. The participants agreed that the absence of translation services or the use of a problematic language hinders recruitment, which has been highlighted in previous research [19]. Also, participants emphasised that focusing on the medical perspective has hindered researchers from considering the social aspects. The emphasis on the medical aspect has restricted the exploration of the social perspectives on their conditions, which is crucial for a comprehensive understanding of the condition and transformative thinking about inclusion in research [30].

The lack of capacity was another issue highlighted and is a known education need [31]. When researching vulnerable populations, such as those with rare diseases who may lack decision-making capacity, there are numerous ethical and legal considerations, especially regarding obtaining informed consent to participate in a study. This will likely involve education needs around these complex legal and ethical issues.

The open-text comments and non-scaled responses indicate that there is recognition of change. However, not enough progress has been made to ensure that the rare disease community feels represented at all phases of research, including conception, assessment, analysis, dissemination, and support of previous findings on creating inclusive research for underserved communities [32,33]. This is succinctly represented in the quote from an advocate who also has a rare disease diagnosis: “Research that involves and considers participants in the design, deliver monitoring and evaluation throughout …”. Advocates and those with rare diseases can support stakeholders in designing, participating, and disseminating findings.

International research initiatives are enhancing participation and treatment in rare disease research. This includes the National Institutes of Health (NIH) Rare Diseases Research and Patient-Centered Research Institutes (PCORI) in the United States, along with the Canadian Institutes of Health Research (CIHR) and the European Reference Networks (ERNs) [34,35,36,37]. The UK has significantly incorporated public and patient involvement (PPI) in research [38,39]. Health and social care research funders require PPI in research applications. It goes some way to ensure research relevance and impact. Healthcare workers potentially have a unique role in PPI engagement and addressing inequity in underserved communities’ access. Collaborations between the public and private sectors, including organisations like the NIH, industry partners, academic institutions, and patient advocacy groups, have been shown to be effective in combining resources and research. This might suggest that nurses, with their person-centred training, research knowledge and understanding of healthcare systems, are well positioned to identify and address the barriers and disparities in research inclusion and engagement experienced by certain sectors of society [40].

## 5. Limitations

The study gathered data from charity representatives, advocates, and individuals with rare diseases. However, the online survey responses were limited due to short recruitment timelines, budget constraints, potential gatekeeper bias as the population is perhaps already engaged and social desirability bias associated with self-reported surveys. Compensation for the participants’ time could improve the response rates and inclusion. The sample size was small, so it is challenging to generalise the results, and more extensive studies are necessary to assess the reliability and validity of the findings in detail. Ethnicity was not collected, as it is not part of the standard SOGI questionnaire; this needs to be addressed in future research. The convenience sampling used in this study is prone to selection bias and is affected by factors beyond the researchers’ control. The authors cannot make broad conclusions based on the responses provided, as the specific feedback received does not represent the views of the entire community. While the feedback is valuable from an individual perspective, it does not offer insights applicable to the larger population. The study implemented strategies like working with established gatekeepers to engage with diverse rare disease communities. It is recognised that the final sample needs to be more diverse regarding rare diseases. However, this rare disease community-led survey offers a glimpse into the wishes, experiences and awareness of perceived barriers to accessing research, highlighting the misconceptions and biases that must be addressed by educating researchers, healthcare professionals, and advocates in the rare disease space.

## 6. Conclusions

The present findings extend the scope of such research by focusing on participation in all types of research and with a focus on the rare disease community. The study uncovers several complex issues around the low levels of diverse participation and inclusion in research: overcoming these barriers holds the potential to develop new treatments for the groups that are most in need and under-resourced, offering hope and optimism for the future; a list of actionable recommendations based on the findings regarding participation and inclusion in research includes fostering trust through transparent engagement and collaboration (row 5, Table 3); providing research materials and communication in multiple languages to reach a broader audience (rows 1 and 7, Table 3); offer financial assistance or compensation for participation in research to alleviate economic barriers that may prevent involvement (rows 4 and 6, Table 3); establish cultural frameworks to address identified barriers to participation and ensure the protection of vulnerable populations, as well as guidance for including adults who may lack standard consent processes in research (rows 2 and 3, Table 3).

As previous research has mainly focused on participation in clinical trials [6,41] rather than wider involvement in the research, the present findings raise several significant concerns regarding the diversity, equality, equity, and inclusiveness of the rare disease community in research and further contribute to the literature by focusing the sample on the rare disease community’s views and opinions of research inclusion. With their understanding of rare disease community, rare disease advocates are well-placed to work with patients and advocate against the disparities and barriers to achieve access for those segments of society. These findings indicate a need for healthcare researchers to work with those in the rare disease community diversity in research and to support research access. Improving research inclusion requires trust, research literacy, partnership, and working together in setting the agenda for research. The absence risks research relevance and its potential impact on research practice. Future research should focus on equality by providing equal access to resources and opportunities to participate in research, ensuring that the research process and research ethics address barriers that inhibit those within the rare disease community from participating in research by addressing well-known barriers such as knowledge around capacity to consent, trust, fear, language, and financial considerations for the rare disease community to be part of the research process and research ethics inclusion.

## Figures and Tables

**Table 1:** Characteristics of participants.

Participants Demographics		Number	Percentage
Participant’s gender	Female Male Non-binary Prefer not to say Total	11 5 1 0 17	64.7 29.4 5.9 0.0 100
Participant’s age	18–39 years 40–50 years 51–61 years 62–72 years Total	0 7 5 5 17	0.0 41.2 29.4 29.4 100
Organisation, group, or individual	Patient voice Other * Advocate Charity or 3rd sector Total	5 5 4 3 17	29.4 29.4 23.5 17.7 100
Have you been diagnosed with a rare condition?	No Yes Other (see below) Prefer not to say Total	9 5 2 1 17	52.9 29.4 11.8 5.9 100
Do you have a family member with a rare condition?	No Yes Other (see below) Prefer not to say Total	8 5 2 2 17	47.0 29.4 11.8 11.8 100

Note: The number indicates the number of participants; the percentage is shown to one decimal point rounded to one decimal place. * Other includes NHS, Education, and organisations working with rare diseases (combined to protect anonymity).

**Table 2:** Consideration of economic, diversity, equity, and inclusiveness barriers.

Questions Perceive or Perception on:	Strongly Agree Count (%)	Agree Count (%)	Unsure Count (%)	Disagree Count (%)	Strongly Disagree Count (%)	Mean (95% CI)	Participation Category or Catergories
Q1 Economic: Lack of monetary resources is a barrier to participation.	9 (52.9)	5 (29.4)	1 (5.9)	2 (11.8)	0 (0.0)	M = 4.24 (3.70–4.77)	Advocate
Q2 Funding: Funding applications for research do not always include the support costs.	7 (41.2)	6 (35.2)	3 (17.6)	1 (5.9)	0 (0.0)	M = 4.12 (3.64–5.59)	Charity, patient groups and others
Q3 Intersectionality: Researchers do not always understand what it is and what it does.	6 (35.2)	7 (41.2)	2 (11.8)	2 (11.8)	0 (0.0)	M = 4.00 (3.49–4.51)	Charity and others
Q4 Access: Researchers are often unsure about how to access participants from diverse groups.	9 (52.9)	5 (29.4)	2 (11.8)	1 (5.9)	0 (0.0)	M = 4.29 (3.82–4.77)	Charity and others
Q5 Cognition: Studies do not always demonstrate how to include participants who may lack capacity.	8 (47.0)	7 (41.2)	0 (0.00)	2 (11.8)	0 (0.0)	M = 4.24 (3.74–4.73)	Charity
Q6 Cultural competence: Research teams often lack the understanding on how it may create a barrier to participation.	9 (52.9)	3 (17.6)	1 (5.9)	4 (23.5)	0 (0.0)	M = 4.00 (3.34–4.66)	Advocate and others
Q7 Language: The use of complicated language or the lack of translation is a barrier to participation.	7 (41.2)	9 (52.9)	0 (0.0)	1 (5.9)	0 (0.0)	M = 4.29 (3.90–4.69)	Patient groups
Q8 Disability: Very often, the medical model of disability prevents researchers from seeing the social model of disability.	7 (41.2)	6 (35.2)	2 (11.8)	2 (11.8)	0 (0.0)	M = 4.06 (3.53–4.59)	Advocate
Q9 Psychosocial: Issues such as anxiety, fear, safety and trust are a barrier to research participation.	7 (41.2)	10 (58.8)	0 (0.0)	0 (0.0)	0 (0.0)	M= 4.06 (3.53–4.59)	Patient groups

Note: The number indicates the number of participants, and the number in parenthesis is the percentage. Interpretation of 5-point Likert scale, strongly agree 4.20–5.00, agree 3.40–4.19, unsure 2.60–3.39, disagree 1.80–2.59, strongly disagree 1.00–1.79.

**Table 3:** Examples of joint display of integrated data collection for convergent mix method design.

Quantitative Data	Data Source and Item	Qualitative Data	Data Source	Initial Codes and Theme
The use of complicated language or the lack of translation in the recruitment process is a barrier to research participation.	ST Q7	“I have never worked at, or even heard about, a research institution that provided language translation services to enable research. Puzzlingly, many of these institutions also teach languages”	Other, with a rare condition, 40–50 age group, male.	Codes: Language, translation
Research teams often lack the understanding of how different cultural perspectives may create a barrier to participating in research.	ST Q6 and Q5	“tend to stay with the same people and its easier”	Advocate, no rare condition, 51–61 age group, female.	Theme: Accessibility and language in research
Researchers do not always understand what intersectionality is and what intersectionality does. The medical model of disability prevents researchers from seeing the social model of disability.	ST Q3	“… all included in the research process to ensure these voices are heard. I believe this type of research seeks to take these voices into the overall conversation and research design”	Advocate, with a rare condition, 40–50 age group, female.	
Funding applications for research do not always include the support costs associated with the additional time or resources required to include participants from under-represented diverse rare disease communities.	ST Q1 and Q2	“Research that involves and considers participants in the design, deliver and monitoring and evaluation throughout”	Advocate with a rare condition, 62–72 age group, female.	
Issues such as anxiety, fear, safety and trust are a barrier to research participation.	ST Q9	“Fears of harm, fear of stigma, fear of unemployment, fear of side effects, these are all very real.”	Other, with a rare condition, 40–50 age group, male.	Codes: Respect and autonomy, trust and beneficence, bias and misconception
Economic poverty (refers to lack of monetary resources to meet needs) is a barrier to research participation.	ST Q1	“Research which aims to take into consideration the diversity of the population to study and aims to include underrepresented groups in the study.”	Patient or patient group, no rare condition, 40–50 age group, male.	Theme: Tensions involved in achieving participation
Researchers are often unsure about how to access participants from diverse groups.	ST Q4 and Q8	“tend to stay with the same people and its easier”	Advocate, no rare condition, 51–61 age group, female.	

## Data Availability

Data are provided within the manuscript text and tables. The data used to support the findings of this study are also available from the corresponding author upon reasonable request.

## Public Involvement Statement

Members of the public (advocates, charities and organisation supporting rare diseases) were involved as active partners in the survey questions, design and data collection.

## Guidelines and Standards Statement

This manuscript was drafted with the STROBE (Strengthening the Reporting of Observational Studies in Epidemiology) guidelines for reporting observational research.

## Abbreviations

The following abbreviations are used in this manuscript:

EDIRA	Equality, Diversity, and Inclusive Research Association
EEDI	Equality, Equity, Diversity and Inclusion
EDI	Equity, Diversity and Inclusion
DEI	Diversity, Equity and Inclusion
